# Childhood tuberous sclerosis complex in southern Sweden: a paradigm shift in diagnosis and treatment

**DOI:** 10.1186/s12887-023-04137-4

**Published:** 2023-06-29

**Authors:** Kevin Pearsson, Josefin Björk Werner, Johan Lundgren, Lotta Gränse, Emma Karlsson, Kristina Källén, Erik A. Eklund, Zivile Bekassy

**Affiliations:** 1 Department of Clinical Sciences Lund, Clinical Sciences Helsingborg, Lund University, Lund, Sweden; 2 Department of Paediatrics, Helsingborg General Hospital, Helsingborg, Sweden; 3 Section for Paediatric Cardiology, Skåne University Hospital, Lund, Sweden; 4 Section for Paediatric Neurology, Skåne University Hospital, Lund, Sweden; 5 Department of Ophthalmology, Skåne University Hospital, Lund, Sweden; 6 Department of Paediatrics, Blekingesjukhuset, Karlskrona, Sweden; 7 Pediatrics, Clinical Sciences Lund, Lund University, Lund, Sweden; 8 Section for Paediatric Nephrology, Skåne University Hospital, Lund, Sweden

**Keywords:** Angiomyolipoma, Astrocytic hamartoma, Cardiac rhabdomyoma, Epilepsy, Everolimus

## Abstract

**Aim:**

To investigate the complete clinical spectrum of individuals with paediatric tuberous sclerosis complex in southern Sweden and explore changes over time.

**Methods:**

In this retrospective observational study, 52 individuals aged up to 18 years at the study start were followed-up at regional hospitals and centres for habilitation from 2000 to 2020.

**Results:**

Cardiac rhabdomyoma was detected prenatally/neonatally in 69.2% of the subjects born during the latest ten years of the study period. Epilepsy was diagnosed in 82.7% of subjects, and 10 (19%) were treated with everolimus, mainly (80%) for a neurological indication. Renal cysts were detected in 53%, angiomyolipomas in 47%, astrocytic hamartomas in 28% of the individuals. There was a paucity of standardized follow-up of cardiac, renal, and ophthalmological manifestations and no structured transition to adult care.

**Conclusion:**

Our in-depth analysis shows a clear shift towards an earlier diagnosis of tuberous sclerosis complex in the latter part of the study period, where more than 60% of cases showed evidence of this condition already *in utero* due to the presence of a cardiac rhabdomyoma. This allows for preventive treatment of epilepsy with vigabatrin and early intervention with everolimus for potential mitigation of other symptoms of tuberous sclerosis complex.

## Introduction

Tuberous sclerosis complex (TSC) is a rare autosomal dominant disorder with almost complete penetrance that affects multiple organ systems [1]. The estimated prevalence of TSC in Sweden is 5.38/100 000 individuals [2]. TSC is caused by loss-of-function variants in tumour suppressor genes either *TSC1* or *TSC2*, which cause upregulation of the mechanistic target of rapamycin1 (mTOR1) signalling pathway [3]. In TSC, mTOR1 signalling pathway disinhibition accelerates cell growth and proliferation, with subsequent growth of hamartomatous tumours in the brain, heart, kidneys, eyes, skin, and lungs [4]. TSC can be diagnosed based on clinical diagnostic criteria, or by finding a pathogenic *TSC1 or TSC2* variant, or both [1].

In the last few decades, TSC diagnostics, treatment, and surveillance have advanced markedly [1, 5]. For instance, everolimus, an mTOR-inhibitor (mTORi), has been found particularly efficient for treating subependymal giant cell astrocytomas (SEGA), angiomyolipomas (AML), facial angiofibromas, and epilepsy in TSC patients, if used continuously [6–9].

Focal seizures and infantile spasms often occur in the first year of life [2, 4] and early seizure onset is associated with intellectual disability (ID) and drug-resistant epilepsy (DRE) in TSC patients [10]. Preventive treatment with vigabatrin, introduced at the first detection of epileptiform activity, before clinical seizure onset, may delay epilepsy onset and decrease its severity [11].

We reviewed the complete TSC clinical spectrum in a Swedish paediatric cohort and investigated changes in diagnostics and treatment over the last two decades. To our knowledge this is the first not registry-based retrospective study of all TSC manifestations in the modern era.

## Methods

### Patients and setting

All individuals younger than 18 years at the beginning of, or who were born during the study period (January 2000 to December 2020), fulfilling TSC diagnostic criteria, and followed up at the hospitals and centres for habilitation of children and adolescents in southern Sweden (Skåne, Kronoberg, and Blekinge counties), were included. Collected data included demographics, family history of TSC, genetics, clinical manifestations, radiological findings, and treatment. Medical records were reviewed by specialists in the respective areas.

Available echocardiographs and electrocardiograms (ECG) were reviewed for cardiac rhabdomyomas (CRs) and arrhythmias, respectively. The largest CR diameter on the earliest echocardiographic examination was recorded.

From brain images, taken from radiology reports, we recorded cortical tuber, subependymal noduli, SEGA, and hydrocephalus data. The earliest scans were obtained by computed tomography (CT), which was later replaced by magnetic resonance imaging (MRI). SEGA was defined as a caudothalamic groove lesion with a size > 1 cm in any direction, or any subependymal lesion that showed growth on serial imaging, regardless of size [12]. DRE was defined as continuous disabling seizures despite having trialled at least two appropriate antiseizure drugs (ASD) at adequate doses [13]. DRE presence was investigated in four different age-spans: < 5, 5 to < 10, 10 to < 15, and 15–18 years of age. If DRE occurred in any age-span, the individual was defined as having DRE in that period; however, not until having reached the upper limit of the age-group. Data were retrieved regarding epilepsy surgery and included: (1) discussions among colleagues, without further referral to a specialist team, (2) discussions only with the parents, without further referral to a specialist team, and (3) results from structured epilepsy surgery management rounds. We further recorded whether the individual had undergone epilepsy surgery, was accepted for surgical evaluation, or was deemed ineligible for surgery, and if the parents had declined epilepsy surgery despite having been offered this treatment.

Intellectual disability (ID) and neuropsychiatric (NP) disorders were diagnosed using standard psychological testing material. Non-NP linguistic problems were noted. Motor impairment was judged by physiotherapists.

Renal imaging data were taken from the radiology reports and presence of AML and cysts recorded. First and last available creatinine values, urine dipstick results, and blood pressure, if two or more values were available, were recorded. Hypertension was defined as systolic and/or diastolic blood pressure ≥ 95th percentile for sex-, age-, and height-matched children.

Ophthalmological records were reviewed for fundus appearance, electroretinography (ERG), and cerebral visual impairment (CVI). Due to the large spread in age at last available ophthalmologic examination, visual acuity was not compared, as the visual system is not fully developed in growing children.

### Statistical method

Data were managed using the Research Electronic Data Capture database hosted by Lund University, Sweden, and were analysed using SPSS v27 software (IBM Corp., Armonk, NY). Data are presented as median and range. Differences between groups were assessed by the Mann–Whitney *U* test for continuous variables and Pearson Chi-square or Fischer´s exact test, as applicable, for two dichotomous variables. The level of significance was set at p < 0.05.

### Ethical consideration

The study was approved by the Swedish Ethical Review Authority and performed in accordance with the declaration of Helsinki.

## Results

Characteristics and genetic findings of study individuals are presented in Table 1.

**Table 1:** Demographics and genetics of the study individuals

Total number	52
Female	25 (48%)
Age < 18 years at the end of the study period	27 (52%)
Age at the end of the study period, median (range), years	17 (0.0–37.0)
Heredity	6 (11.5%): maternal 4, paternal 2
Genetics	*TSC1* 10 (19.23%) *TSC2* 11 (21.15%) *TSC2/PKD1* 1 (1.92%) NMI in *TSC2*, *TSC1* not analyzed Mosaicism 1 (1.92%) *PTPN11* 1 (1.92%) NMI 3 (5.76%) Not tested 26 (50%)

NMI: no mutation identified; *PTPN11* 1 mutation characterising Noonan syndrome was found in a patient fulfilling criteria for TSC

### Cardiac manifestations

**Fig. 1:**
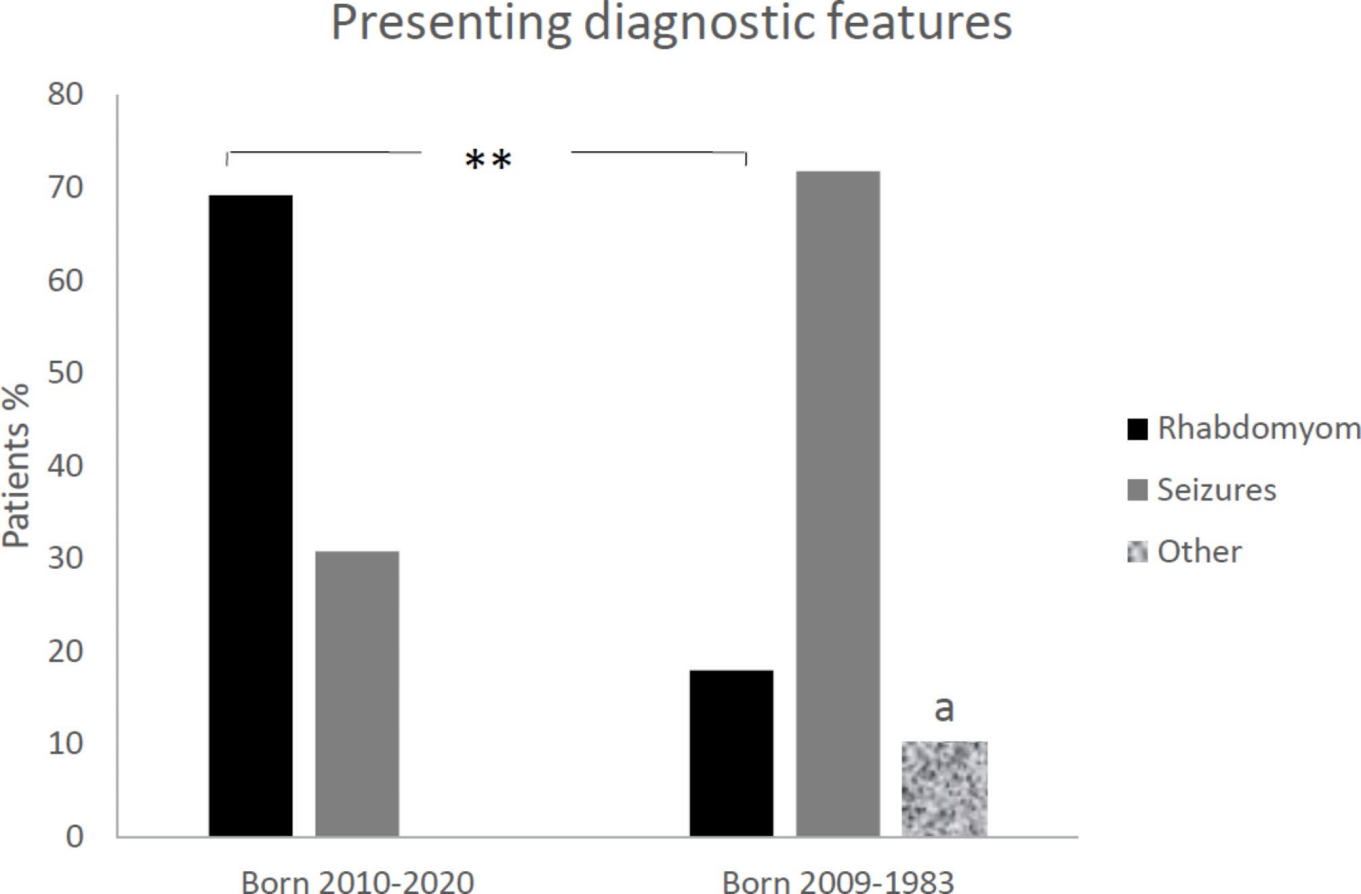
**Comparison of presenting diagnostic features in tuberous sclerosis complex individuals in two periods** **a**) palpable kidneys, shagreen patches, astrocytic hamartoma, and headache in one patient each. **P < 0.01

**Fig. 2:**
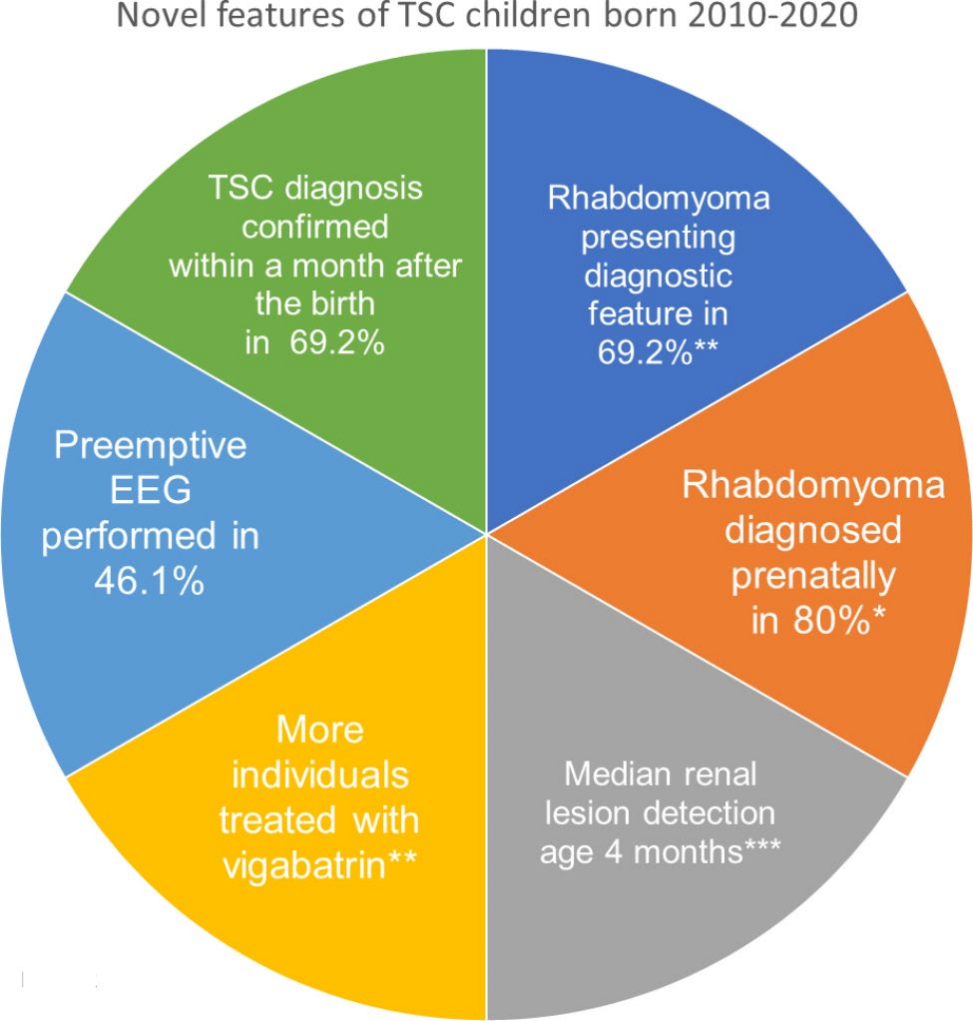
Novel features of TSC children born 2010–2020 Summary of the most important changes in the practical care of children born 2010–2020 compared to 1983–2009. *P < 0.05, **P < 0.01, ***P < 0.001 EEG: electroencephalography, TSC: tuberous sclerosis

In the total cohort, CRs were detected in 77.5% (31/40) of the individuals, of which 38.7% (12/31) involved prenatal detection. As a feature leading to early diagnosis, CRs were significantly more often found in individuals born during 2010–2020 than in those born during 1983–2009 (p = 0.001) (Fig. 1). Furthermore, 9/13 (69.2%) individuals born during 2010–2020 presented with CRs in the neonatal period and the TSC diagnosis was confirmed within a month (Table 2; Fig. 2). There were significantly more individuals diagnosed prenatally of those born after (80%, 8/10) than before (33%, 4/12) 2010, (p = 0.043). Cardiac manifestations are presented in Table 3. No other cardiac manifestation than rhabdomyomas had a significant difference before and after 2010.

**Table 2:** Other diagnostic features, and epilepsy-related manifestations, treatment, and follow-up, of patients presenting with rhabdomyoma 2010–2020

ID #	Diagnosis confirmed		Mutation	EEG monitoring	Vigabatrin treatment			MRI		Last follow-up			
	Age	By			Start	EEG	Seizures after start	Initial	Follow-up	Age	Seizures	EEG	Medication
1	At birth	Heredity, Hypo- melanotic macules	*TSC2*	Yes	Preventive at 4.5 months	IEA	No	At 11 months: T, SEN, suspected SEGA	At 1.5 years unchanged	1.5 years	No	N	VGB
2	1 month	Genetics	*TSC2*	Yes	After febrile focal seizures at 9 months	N	No	At 1.5 years: T, SEN	Not performed	1.5 years	No	N	VGB
3	1 month	MRI brain	NMI	Yes	After focal seizures at 4 months	SA	Yes	At 1 month: T, SEN	At 4 years unchanged	4.5 years	DRE	IEA	VGB TPM OXC VPA
4	3 weeks	MRI brain	*TSC2*	Yes	No	N	NA	At 3 weeks: T, SEN, suspected SEGA	At 3 years 10 months unchanged T, SEN, no SEGA	5 years 8 months	No	-	-
5	1 month	MRI brain	NMI	Yes	Preventive at 4 months	IEA	Yes	At 1 month: T, SEN	At 5.5 years unchanged	6.5 years	No	SA	VGB tapering VPA
6	1 week	MRI brain	Not tested	No	At 2 years and 4 months	IEA	Yes	At 1 week: T, SEN	At 16 months T, SEN, abnormal cortex frontal lobe	5 years 7 months	No	IEA	No ASD, seizure free after epilepsy surgery
7	1 week	US brain and kidney cysts	NMI (only *TSC2* analysed)	Yes	After focal seizures at 5 months	IEA be- fore seizu- res	Yes	At 2 months: T, SEN, suspected SEGA	At 5 years 1 month progress of T, SEN; SEGA unchanged	7 years	Rarely	IEA	Ketogenic diet
8	1 month	Hypome- lanotic macules	*TSC1*	No	After focal seizures at 6,5 years	IEA	Yes	At 4 months: T, SEN	At 3 years 7 months unchanged	9 years 7 months	Weekly	IEA	Everolimus OXC
9	1 week	Hypome- lanotic macules, astrocytic hamartoma, kidney cysts	*TSC2*	No	After focal seizures at 13 months	IEA be- fore seizu- res	No	At 11 months: T, SEN	At 8 years 5 months unchanged	10 years 4 months	No	N	TPM CLB

EEG: electroencephalography; MRI: magnetic resonance imaging; IEA: interictal epileptiform activity; T: tubers, SEN: subependymal noduli; SEGA: subependymal giant cell astrocytoma; VGB: vigabatrin; N normal; NMI: no mutation indentified; SA: seizure activity; DRE: drug-resistant epilepsy; TPM: topiramate; OXC: oxcarbazepine; VPA: valproic acid; NA: not applicable; ASD: anti-seizure drugs; US: ultrasound; CLB: clobazam

**Table 3:** Cardiac manifestations in tuberous sclerosis patients

Variable		Available records
**Rhabdomyomas** n (%)	31 (77.5%)	40
**Rhabdomyoma size, mm** Median (25th & 75th percentile)	10 (7–20)	20
**Foetal diagnosis** n (%)	**12 (38.7%)**	31
Rhabdomyoma	7 (22.6%)	
Decreased ventricular function	1 (3.2%)	
Rhabdomyoma + arrhythmia	3 (9.7%)	
Rhabdomyoma + arrhythmia + decreased ventricular function	1 (3.2%)	
**Affected cardiac function** n (%)	**4 (11.4%)**	35
Decreased ventricular function	1 (2.9%)	
Valvular obstruction	2 (5.7%)	
Decreased ventricular function + valvular obstructions	1 (2.9%)	
**Postnatal arrhythmia** n (%)	**4 (11%)**	36
Supraventricular tachycardia	2 (5.5%)	
Supra- and ventricular tachycardia	1 (2.8%)	
Unspecified tachycardia	1 (2.8%)	
**ECG abnormality** n (%)	**8 (23.5%)**	34
Pre-excitation	5 (14.7%)	
Complete right bundle branch block	1 (2.9%)	
ST-abnormality	2 (5.9%)	
**Regression** n (%)		20
Complete	9 (45%)	
Partial	7 (35%)	
Not specified	4 (20%)	
**Follow up** n (%)		30
At paediatric cardiologist		
Ongoing	10 (33.3%)	
Terminated	10 (33.3%)	
Referred to adult cardiologist Yes No	4 (13.3%) 6 (20%)	

One boy with an intrauterine tentative diagnosis of CRs had pre- and postnatal tachycardia. Postnatal echocardiography confirmed multiple CRs localized in the right atrium and both ventricles, close to the tricuspid valve, completely obstructing the right ventricular (RV) inflow. He received a Blalock–Taussig shunt at 3 days old, which was catheter-closed at 11 months, due to CR regression and pulmonary over-circulation. Another boy had an intrauterine diagnosis of cardiac tumours and supraventricular tachycardia (SVT). A postnatal ECG displayed pre-excitation and SVT and he was treated with digoxin until 1 year old. The largest tumour obstructed RV outflow, necessitating surgical resection at age 1 week. Most CRs underwent spontaneous involution, but at least one remained detectable by 18 years old. Ventricular function was affected in two new-borns: in one, tumour masses occurred in both ventricles and filled the RV, although not necessitating any operative or medical treatment.

### Neurological manifestations

Cortical tubers and subependymal nodules were found in 49 and 48/51 subjects (1/52 did not undergo CT or MRI). One individual that showed tubers, but not subependymal noduli, exhibited a mild phenotype with skin lesions and retinal astrocytic hamartomas, without clinical signs of neurological disease.

SEGA was identified in 16/51 (31.4%) individuals. Surgical treatment was performed in four and everolimus treatment was used in seven individuals (Table 4). Hydrocephalus was reported in four, with missing data in 15.

**Table 4:** Everolimus (EVE) treatment

ID #	Indication	Age at start	Duration	Mean conc ng/mL	Effect on epilepsy					Effect on SEGA (tumour volume)	Effect on kidney manifestations	Astro cytic hamar tomas (AH)	Side effects
					Seizures	EEG		Medication					
					After 3 months EVE	Before	After 1 year EVE	Before	During EVE^a^				
1	Seizures SEGA	5.3 years	9.6 years	6.3	Decrease > 90%	CSWS	Focal IEA frequent	CLB TPM ESM	CLB ZON	Decrease > 50%	AML stable Cysts growing	NA^b^	Gingivitis Increased TG
2	Seizures	2.10 years	7.8 years	6.4	Decrease < 50%	IEA frequent	No change	VPA TPM LEV	CLB VGB	No further growth	Decrease of single cyst	NA	Increased TG
3	Seizures	11.2 years	6.10 years	7.2	Decrease < 50%	IEA frequent	No change	LTG LEV CLB PB	LEV CLB PB STM PER	No further growth	NA	NA	Increased TG
4	Seizures SEGA	13 years	5 years	4.5	Decrease < 50%	IEA sparse	No change	LTG TPM Pyridoxin	LTG Pyridoxin	Decrease < 50%	No change of AML	AH appears during treat- ment	No
5	AML	8.6 years	3.10 years	6.0	No seizures	NA	NA	NA	NA	No further growth	Decrease 50% of biggest AML Decrease of cysts	No change	Gingivitis VZV reacti- vation Increased TG
6	Seizures	14.11 years	3 years	6.1	Decrease > 50%	No EEG	IEA sparse	OXC LTG	OXC LTG	No further growth	No change of cysts	NA	No
7	Seizures	7.7 years	2.2 years	4.1	Decrease > 90%	IEA frequent	No change	OXC VGB VPA	OXC	NA	No change of single cyst	NA	Gingivitis
8	Seizures	12.6 years	1.11 years	1.6	Decrease > 50%	-	-	LTG VPA CLB PER	LTG VPA CLB PER	No further growth	No change of multiple bilateral minimal AML	NA	No
9	AML	13.10 years	1 year inter- mittent	NA^c^	NA	NA	NA	NA	NA	NA	NA^c^	NA^c^	Gingivitis
10	Seizures IS	4.5 months	4 months	4.6	Seizure free	IEA	Normal	VGB	VGB	NA	NA^d^	No AH	Increased TG

NA: not applicable. SEGA: subependymal giant cell astrocytoma; EEG: electroencephalogram; ^a^During everolimus treatment at last follow-up; CSWS: epilepsy with continuous spike-wave during slow wave sleep; IEA: interictal epileptiform activity; ESM: ethosuximide; CLB: clobazam; TPM: topiramate; ZON: zonisamide; AML: angiomyolipoma; NA^b^: this patient underwent enucleation of the eye before start of everolimus treatment; TG: triglycerides; VPA: valproic acid; LEV: levetiracetam; LTG: lamotrigine; VGB: vigabatrin; PB: phenobarbital; STM: sulthiame; PER: perampanel; VZV: varicella zoster virus; OXC: oxcarbazepine; NA^c^: this patient stopped the treatment in several occasions because of the gingivitis, the evaluation of the effect was impossible due difficulties to diagnose AML in polycystic kidneys; IS: infantile spasm; NA^d^ : no repeat renal imaging was performed. Rhabdomyomas not included due to spontaneously regression during childhood

Forty-three individuals (82.7%) had an epilepsy history. Median age at seizure onset was 11 months (range 2–85 months). Twenty-three individuals developed epilepsy before the age of 1 year, of which 15 had infantile spasms. EEG showed hypsarrythmia in 12/15 individuals. Focal seizures were present 1–8 months before infantile spasm onset in 3/15 patients. These spasms developed to seizures with focal onset, either directly, or after a spasm-free period, with a semiology related to the seizure-onset zone. Three individuals had absences and drop attacks suggestive of Lennox–Gastaut syndrome.

Of all individuals with epilepsy, 27/43 (62.8%) had DRE in at least one age-span (Table 5). Among the 25 individuals who turned 18 years old during the study period (thus completing all age-spans), four (16.0%) were never diagnosed with epilepsy and three (14.3%) never developed DRE, whereas seven (33.3%) had DRE across all age-spans. Six (28.6%) of the 25 individuals did not have complete data for all age-spans.

**Table 5:** Data concerning tuberous sclerosis epilepsy surgery by age spans

	< 5 years	5 – <10 years	10 – <15 years	15 − 18 years
Presence of drug-resistant epilepsy, n (% of all epilepsy cases)	20 (56)	14 (42)	12 (48)	11 (55)
Epilepsy surgery never discussed, n (%)	8 (40)	5 (35.5)	6 (50)	8 (72.7)
Epilepsy surgery performed, n (%)	3 (15)	1 (7.1)	1 (8.3)	0
Surgery declined at specialist-team management round, n (%)	2 (10)	2 (14.3)	1 (8.3)	1 (9.1)
Interrupted surgery evaluation, n (%)*	1 (5)	1 (7.1)	1 (8.3)	1 (9.1)
Eligible for surgery, declined by parents, n (%)	2 (10)	1 (7.1)	2 (16.7)	0
Discussed among colleagues and deemed not suitable for referral to specialist-team, n (%)	0	1 (7.1)	0	0
The surgical alternative was discussed only with parents, n (%)	2 (10)	3 (21.4)	1 (8.3)	1 (9.1)
Missing data, n (%)	2 (10)	1 (7.1)	1 (8.3)	0

*Surgical evaluation was interrupted due to seizure frequency decrease or initiation of everolimus

There was no statistically significant difference between those born 1983–2009 and 2010–2020 regarding age of first seizure (p = 0.214), number of patients with infantile spasms (p = 1.0), or number of patients with DRE < 5 years of age (p = 1.0).

Two individuals with West syndrome responded to corticosteroids and nine to vigabatrin treatment. In 1/12 individuals with hypsarrythmia, treatment was unknown. Focal seizures were treated with the following drugs, either alone or in combination: carbamazepine (n = 12), clobazam (n = 11), clonazepam (n = 7), everolimus (n = 10), gabapentin (n = 2), lamotrigine (n = 20), levetiracetam (n = 19), oxcarbazepine (n = 23), perampanel (n = 2), phenobarbital (n = 5), pyridoxin (n = 6), sulthiame (n = 5), topiramate (n = 17), valproic acid (n = 29), vigabatrin (n = 24), and zonisamid (n = 3). Twelve individuals with previous epilepsy were seizure-free at study inclusion, four had discontinued their ASD, and two patients were on monotherapy. In a few individuals, vagus nerve stimulation (n = 2) or ketogenic diet (n = 1) were added, with a marked response. Twelve individuals became seizure-free on ASD monotherapy for more than 2 years. One individual became seizure-free with only a ketogenic diet.

### Everolimus treatment

During 2011–2020, 8/10 individuals were treated with everolimus due to neurological indications (Table 4), the youngest being 4.5 months at treatment start. The longest treatment duration was 9.6 years. The mean everolimus concentration varied from 1.6 to 7.2 ng/mL and was generally well tolerated, with gingivitis and hypertriglyceridemia as the most frequently reported side-effects. Two of seven individuals with SEGA showed a tumour size reduction, whereas the rest remained stable. All eight individuals exhibited a decrease in seizure frequency and the number of concomitant ASDs could be reduced in 4/8 individuals.

### Vigabatrin treatment

From 2013, 6/7 individuals with a neonatal TSC diagnosis were followed-up with regular EEG (Table 2; individuals 1–7), four of whom developed interictal epileptiform activity prior to clinical seizures. Preventive vigabatrin was initiated in two individuals, one of whom did not develop clinical seizures. There were significantly more individuals with epilepsy treated with vigabatrin after 2010 (10/11) compared to before (13/32), p = 0.004 (Fig. 2) but no significant difference in age of initiating vigabatrin (p = 0.868).

### Epilepsy surgery discussion

Table 5 presents data on epilepsy surgery in individuals with DRE and reasons for not proceeding with surgery. Epilepsy surgery was performed in only 5/43 individuals with DRE. Among individuals with DRE across at least two age-groups (n = 14), surgical treatment was never discussed in only one. Among individuals with DRE in only one age-span of DRE, 6/13 never discussed epilepsy surgery. Discussions concerning epilepsy surgery most frequently involved individuals in the age-span 5 to < 10 years (9/14, 64.5%). In a few cases, surgical evaluation was interrupted due to seizure frequency decrease or the initiation of everolimus.

For those born 1983–2009, 4/13 had a discussion about epilepsy surgery before 5 years of age compared to 4/5 for those born 2010–2020 (p = 0.118).

### Neuropsychiatric and motor disorders

Normal cognitive function was observed in 23/52 (44%) individuals. Thirteen showed mild, 14 moderate, and two severe ID. Attention deficit disorder (ADHD) was mild in seven and moderate in three, and autism was mild to moderate in 13, and severe in seven. Language impairment, not attributed to ID, autism, or ADHD, was reported in seven individuals, and was severe in two. Neuropsychiatric and cognitive symptoms, collectively known as TAND, were very common in our cohort, but was rarely formally reported in the patient chart. Motor impairment was recognised in 11 (22%) individuals.

An ordinary school program was attended or finished by 23 (44%; two with special support), 20 (38%) were in special schools for individuals with ID, two (4%) had not started school, and data were missing for seven (13%).

### Renal manifestations

Appropriate renal imaging was available for 49/52 individuals. Eleven individuals underwent repeat ultrasound (US) examinations, and the remaining individuals underwent both US and MRI or occasionally CT and urography. Median follow-up was 4.83 years (0.25–16.67 years). Median renal lesion-detection age for individuals born during 2010–2020 was 4 months vs. 116 months for those born 1983–2009 (p = 0.0001, Fig. 2). No renal lesions were observed in 6/22 (27.3%) individuals who had passed 18 years of age, and in 4/27 (14.8%) who were younger than 18 years. No renal cell carcinoma was found in this cohort.

AMLs were found in 23 (47%) individuals. These were single in three, multiple unilateral in four, and multiple bilateral in the remaining individuals. Two individuals underwent embolization of asymptomatic large AMLs before everolimus introduction. In one girl, everolimus treatment significantly reduced AML size, as well as multiple small renal cysts. The effect on renal manifestations in individuals treated with everolimus for neurological indications is presented in Table 4.

Renal cysts were detected in 26 (53%) individuals: these were single in five, multiple unilateral in one, and multiple bilateral in the remainder. Contiguous deletion of *TSC2/PKD1* was found in one individual, who presented with renal cysts at age 6 months, hypertension at 14 months, and developed a clinical course akin to autosomal dominant polycystic kidney disease. Everolimus treatment was initiated at age 14 years, due to suspicion of AMLs, but was discontinued because of side effects and uncertain benefits. She developed end-stage renal disease and underwent successful kidney transplantation at age 17.5 years. The right kidney removed on this occasion contained multiple AMLs.

Eleven individuals (22.4%) exhibited both renal cysts and AMLs. Small hyperechoic lesions in six individuals, considered as AMLs on US by a radiologist, were interpreted as renal cysts on MRI. In all eight individuals with CRs identified prenatally, renal lesions were detected during the first year of life. Small lesions could not be defined in 3/5 individuals who underwent US during the first month of life. Follow-up investigations showed renal cysts in all these individuals.

Kidney function tests were available for 37 (71%) and blood pressure measurements for 17 (32.6%) individuals. Only the individual with the *TSC2/PKD1* deletion was diagnosed with hypertension and decreased kidney function. Urinalysis was available in 15 (28.8%) individuals. Intermittent microscopic haematuria was found in four (one with AML, two with renal cysts and one with no lesions on renal imaging), and proteinuria in one. No proteinuria was found in individuals treated with everolimus.

Renal data were available for 22/25 individuals at 18 years. Ten individuals were transferred for follow-up care under an adult urologist or nephrologist, four to primary care, and two to a neurologist. One individual without renal manifestations was transferred to primary care while the remaining five had no planned follow-up.

### Hepatic manifestations

One individual with renal cysts had hepatic cysts. AMLs in both the kidneys and the liver were detected in three individuals. One patient had suspicious, non-defined liver lesions.

### Ophthalmological manifestations

Ophthalmological records were available for 50 individuals. At the last registered ophthalmological examination, the median age was 13 (range 1.3–29.7) years. Astrocytic hamartomas in one or both eyes were detected in 16/50 (32%) individuals, and achromatic patches in 4/50 (8%). One individual developed a growing opticus astrocytoma necessitating eye enucleation, and optic atrophy because of SEGA growth. Vigabatrin treatment was used in 23/52 (44.2%). Full-field ERG was performed in 10 patients. Reduced ERG response was measured in 3/10, resulting in medication reduced or cessation. Some recovery was observed at follow-up examination. No individual was investigated for cerebral visual impairment.

### Dermatological manifestations

In many cases, skin manifestations were not documented in detail in medical records and therefore only treatment is presented. Before age 18 years, 11 (21.2%, three missing data) had laser therapy and 11 (21.2%, two missing data) were prescribed topical rapamycin for facial angiofibromas. Of these, six (11.5%, four missing data) underwent both treatments.

### Pulmonary manifestations

No lung manifestations were reported in this cohort.

## Discussion

As the presenting feature suggestive of TSC, CRs were significantly more commonly detected in individuals born during 2010–2020 than during 1983–2009. A low neonatal diagnostic frequency of CRs in the late nineteen-nineties and early two-thousands has been published previously [14]. A CR was the most common first diagnostic feature (2/3) of children born during 2010–2020, in contrast to seizure onset, which was the presenting TSC feature in > 70% of the older individuals in our cohort. CR led to diagnosis of TSC, within their first month of life, in all children born during the last decade. This is important, as early diagnosis allows identification of emerging problems, such as epilepsy, and provides an opportunity for therapeutic interventions that can potentially alter the disease course [15]. CRs have been diagnosable by foetal ultrasound techniques since 1982 [16], but increasing awareness amongst paediatric cardiologists, improved high-resolution US technologies. Moreover, availability of foetal MRI may explain this significant change during the last 10 years [17].

CRs are usually asymptomatic, but may cause severe morbidity requiring surgical intervention [14], as experienced in two new-borns in this cohort. Pre-excitation was observed in 15% of the individuals, and is a well-known ECG-feature in TSC, where the CR itself is believed to serve as an accessory pathway [18]. Tachycardia was supraventricular in all but one individual, who developed ventricular tachycardia over time. Serial echocardiographic examinations are advised every 1–3 years in the absence of inflow/outflow obstruction and ventricular dysfunction, until the tumour regresses [17]. Thereafter, continued cardiac follow-up with ECG is recommended every 3–5 years [1, 17]. Our study subjects were followed with echocardiography in early life more frequently than these recommendations. However, when cardiac surveillance was stopped, ECG controls also seemed to halt. Echocardiography was the only modality used for cardiac imaging in this retrospective cohort, although MRI is also used [17]. Some case reports indicate successful treatment, with rapid tumour reduction, using both everolimus and sirolimus [19, 20], even for obstructive disease. No patient was treated with mTORi for cardiac indications during the study period; however, we have experience of this as later treatment (Björk Werner, Eklund, personal comm.).

The epilepsy frequency in our cohort (82.7%) agreed with the recently published TOSCA study [21], but was higher than national Swedish data on TSC and epilepsy published by Welin et al. [2]. This may be because epilepsy frequency is overestimated in data from non-population-based studies, as most TSC cohorts, including ours, contain severely affected individuals that require hospital-based care. Furthermore, 34.9% of the epilepsy individuals developed infantile spasms, in line with the TOSCA study [21]. The spasms were the presenting epileptic feature in 80%. Various drugs were used to treat seizures. GABA-potentiating drugs (e.g., valproate, benzodiazepines, barbiturates, and vigabatrin) were predominant (> 40% of prescriptions), similar to the TOSCA study [21]. The use of vigabatrin increased in the most recent decade, in line with recent guidelines [5]. Epilepsy in TSC is often difficult to treat and almost two-thirds had DRE lasting at least one age-span. As epilepsy surgery is the only curative option for DRE, it is noteworthy that half the individuals were never referred for specialist team discussion, thus missing this opportunity. Since 2011, eight individuals were treated with an mTORi for a neurological indication, with an effect both on SEGA growth and seizure frequency. In 4/8 individuals, the number of ASDs could be reduced. ID is often aggravated by DRE. Of our study subjects, 56% fulfilled ID criteria, similar to the findings of the Tuberous Sclerosis 2000 study group [22]. ADHD and autism were also common, but were probably under-appreciated [23], which calls for attention to the related symptoms in daily TSC care.

AMLs and renal cysts were equally observed in this population, as described previously [4, 24]. Renal cysts, however, were found in the first year of life in all individuals with CRs identified prenatally during the latest decade compared to 34,6% of individuals screened for renal lesions in previous decades. Abdominal imaging is mandatory at diagnosis, regardless of age [1]. In our study renal lesions were difficult to define by US during the first month of life. Small AMLs may be hard to distinguish from cysts on US, even in older children, in daily practice [25]. MRI is the preferred modality because of the better structural assessment of the lesions, but requires general anaesthesia in small children and children who are unable to cooperate with the procedure. Progress of renal lesions in our cohort could not be evaluated due to the use of different imaging modalities during the follow-up of the study individuals.

The laboratory analysis and blood pressure measurement showed absence of systematic examination. Both renal cysts and AMLs can cause hypertension and chronic kidney disease with increasing age. Annual assessment of renal function and blood pressure was already recommended in older guidelines [26], and proteinuria assessment has been included in the most recent guidelines [1]. Proteinuria may be a marker of early kidney damage and a potential side effect of mTORi treatment [27, 28]. Everolimus is approved for treatment of asymptomatic growing AMLs > 3 cm in diameter. Some data indicate that mTORi may reduce cyst number and size in individuals with TSC [29], but the effectiveness in TSC-associated renal cystic disease has not yet been fully evaluated. Interestingly, the decreased renal cyst size during everolimus treatment was observed in two individuals in this study.

The occurrence of astrocytic hamartomas and achromatic patches in this paediatric cohort was please merge the text here from the next row somewhat lower than the reported frequency in other cohorts including adults [4]. Of 10 individuals controlled with ERG during vigabatrin medication, retinal effects could be seen in three, with some improvement during the follow-up. Despite involving a small group of individuals, this highlights the importance of closely monitoring these patients for retinal changes. No individual was investigated for CVI problems despite a clear correlation in the literature between neurological ailment and CVI [30]. Ophthalmologists should be encouraged to focus on this diagnosis. Learning ability and quality of life for CVI patients may be substantially improved by a specially trained vision therapist.

Our study has limitations common in retrospective analyses, including incomplete data due to lack of documentation in medical records. No primary healthcare assessment was available and data collection was focused on regional hospitals and centres for habilitation thus some cases with mild disease might have been missed.

## Conclusions

This cohort study of TSC individuals from southern Sweden revealed a paradigm shift in TSC diagnosis, in that the finding of CRs on intrauterine US investigations is often the first sign of TSC, allowing early diagnosis. This allows for preventive treatment of epilepsy with vigabatrin and an early intervention with everolimus for potential mitigation of other TSC symptoms. Despite clear and updated treatment guidelines, we found that adequate follow-up is lacking in some areas, the transition from paediatric to adult care is often insufficiently prepared, and TAND data are often missing. These results confirm the previous proposal on the need for involvement of multidisciplinary teams in TSC care.

## Data Availability

The datasets generated and analyzed during this study is not publicly available as some of these data will be used for another research study. The datasets can be shared from to the corresponding author by reasonable request.

## Abbreviations

AML: Angiomyolipoma
ASD: Antiseizure drugs
ADHD: Attention deficit disorder
CR: Cardiac rhabdomyoma
CVI: Cerebral visual impairment
CT: Computed tomography
DRE: Drug-resistant epilepsy
ECG: Electrocardiogram
EEG: Electroencephalography
ERG: Electroretinography
ID: Intellectual disability
MRI: Magnetic resonance imaging
mTOR1: Mechanistic target of rapamycin1
mTORi: mTOR-inhibitor
NP: Neuropsychiatric
RV: Right ventricular
SEGA: Subependymal giant cell astrocytoma
SVT: Supraventricular tachycardia
TSC: Tuberous sclerosis complex
US: Ultrasound
